# Homeostasis of ammonium and nitrate in plants

**DOI:** 10.1017/qpb.2026.10042

**Published:** 2026-03-27

**Authors:** Yi Chen, Anthony J. Miller

**Affiliations:** Biochemistry and Metabolism, John Innes Centre, Norwich, UK

**Keywords:** ammonium, homeostasis, nitrate, nitrogen, transporter

## Abstract

Nitrogen (N) is a major plant nutrient, and its supply is very often limiting growth. The main forms of inorganic N in soil supplying plants are ammonium and nitrate ions. Although the soil availability of N can vary greatly, the cytoplasmic nutrient ion activities in a typical plant cell are maintained at set points that are independent of changes in supply. By contrast, the storage of N as protein and vacuolar nitrate depends on the external supply. Measurements of cellular homeostasis of ammonium and nitrate are limited by methodology. The upper limits for cytoplasmic set points are likely to depend on toxicity, and for ammonium this is well known but less clear for nitrate. An intracellular set point for N must be maintained by membrane transport systems and assimilation processes. Crop N use efficiency has uptake and assimilation components, and understanding homeostasis is fundamentally important for improving this important trait.

## Graphical abstract

**Figure:**
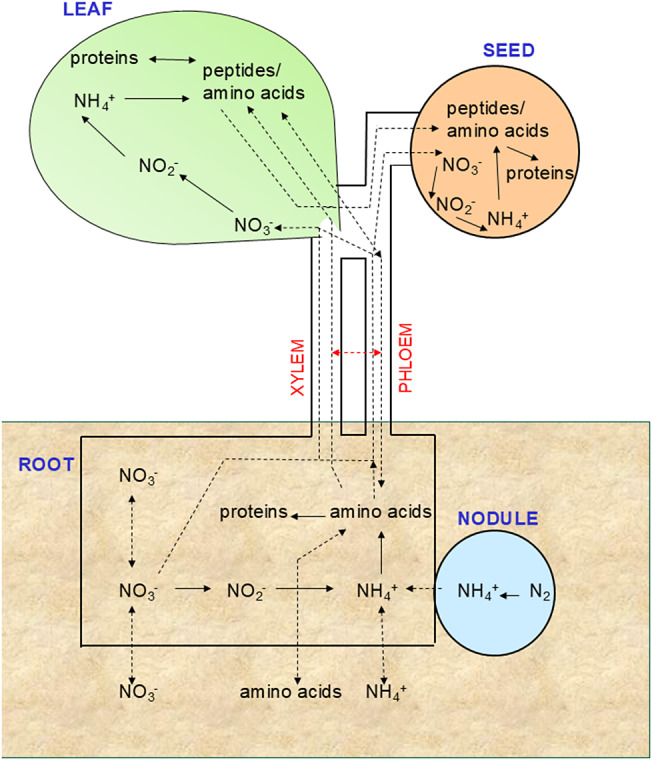

## 1. Introduction

As plants are unable to move to relocate themselves, they need to be able to adapt and adjust to their constantly changing environment. The plasticity of plants is the key to their success, and this requires an ability for plant cells to maintain an internal stability or ‘homeostasis’ even when the external conditions vary widely. Cellular homeostasis requires the preservation of a physiological variable at a set point that is an optimal value for physiology, despite external and internal disturbances. The set point concept comes from control theory, requiring a sensing system that detects deviations from the set point. A regulatory network usually acts via negative feedback to restore the variable towards the set point. In this review, we explore the core idea of homeostasis of plant nitrogen (N), more specifically the inorganic N ions ammonium (NH_4_^+^) and nitrate (NO_3_^−^) within cells. We identify some of the control theory components in plants for NO_3_^−^ and NH_4_^+^ that might define N homeostasis. Transport and assimilation are the main drivers for plant N homeostasis, and the balance between these two processes drives growth and yield. These topics have been extensively reviewed previously, and for brevity, recent overviews are cited.

## 2. Whole plant N homeostasis

**Figure 1.**
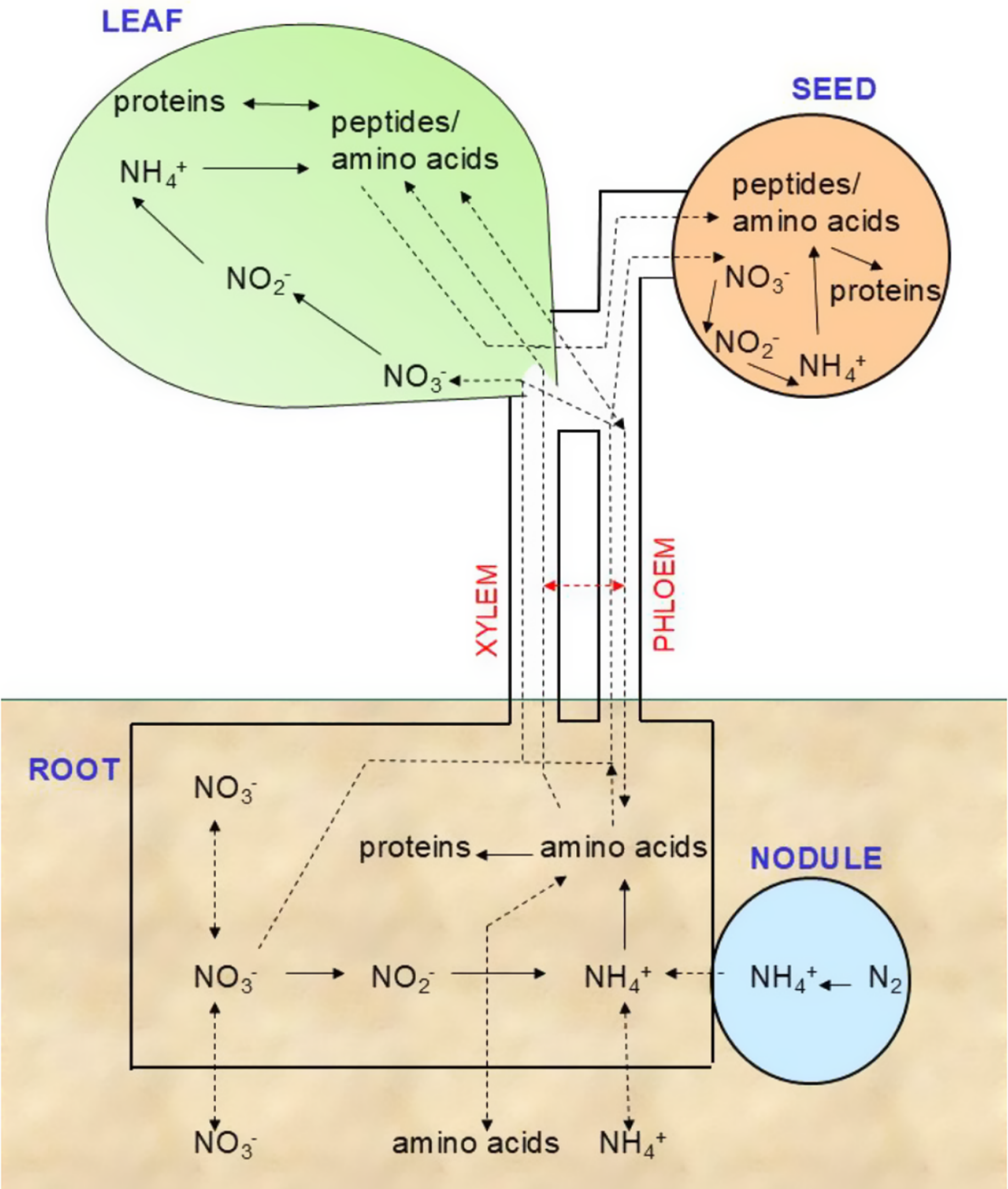
Whole plant N homeostasis pools.

N is an essential macronutrient for plant growth, and the optimisation of agricultural production depends on the addition of N fertiliser. Chemical fertilisers are often applied with N available as NH_4_^+^ or NO_3_^−^, and other forms of organic N, such as urea, are rapidly converted by soil microbes into these inorganic forms. Plants can take up both inorganic N forms: NO_3_^−^ and NH_4_^+^. Legume plants can capture gaseous N_2_ through symbioses with N-fixing microorganisms, forming the neutral molecule ammonia (NH_3_), which can be transferred to the host. Gaseous NH_3_ can be captured by plants and can diffuse through cell membranes, but this environmental source of N is not usually important for growth (Xu et al., 2012). Organic sources of N in the soil, including amino acids, urea and ureides, can be taken up and used by plants. These forms of N are particularly important for plants growing in impoverished soils. N-starved plants have systemic long-distance signalling mechanisms mediated by phloem-mobile microRNAs (miRNAs) (Liang et al., 2012) and peptides (C-terminally encoded peptide or CEP) in the phloem (Ohkubo et al., 2017). Circulating peptide signals are perceived by specific receptors and can modify strigolactone hormone levels to alter root (branching) and shoot (tillering) architecture. In contrast, N-replete status is mediated by circulating cytokinin hormone levels (Poitout et al., 2018). Insufficient N levels can lead to restricted plant growth and a shift from the vegetative to the reproductive developmental phase. Interestingly, N deficiency encourages extensive root development, while sufficiency promotes resource allocation for enhanced vegetative growth (Xu et al., 2012). The process of N allocation is heavily influenced by transporter activity, not only through uptake from the soil but also for the movement of N around the plant. During plant development the distribution of N changes as vegetative growth builds the canopy that later switches to florescence and seed filling (see Figure 1). Throughout these developmental changes the tissue pools of inorganic N ions could alter but only NO_3_^−^ changes directly indicate the overall N status of the plant. For this reason, the crop N status testing applied by farmers uses tissue sap NO_3_^−^, not NH_4_^+^ as an indicator ion species when deciding on fertiliser application rates. This highlights the crucial role of NO_3_^−^ as an indicator ion species and therefore identifies it as a signal in coordinating N utilisation and plant growth. Undeniably, NO_3_^−^ signalling triggers the activation of genes associated with various metabolic pathways and developmental processes (reviewed in Zhao et al., 2018).

Whole plant N homeostasis set points are controlled by variables such as metabolism, growth and signalling to stay balanced. Assimilation, uptake from the soil and long-distance transport within the plant in the phloem and xylem are key processes. The pathways and key enzymes for the assimilation of NO_3_^−^ and NH_4_^+^ are well characterised (Xu et al., 2012). Growth and yield are optimised when N assimilation into amino acids and proteins is most active, and this occurs during periods of maximised photosynthetic carbon assimilation. Nitrate uptake by roots and movement of nitrate to the shoot remains high during the light period and decreases slightly at night. The NO_3_^−^ that is taken up during the nightis chiefly used to replenish the leaf NO_3_^−^ pool (Matt et al., 2001). Once transported to the leaf, NO_3_^−^ is primarily stored within the vacuole, accounting for 58–99% of the total NO_3_^−^ pool (Granstedt & Huffaker, 1982). Therefore, throughout the day and night, the pool of NO_3_^−^ primarily localised in leaf vacuoles, is continually changing and responding to both the soil supply and assimilation activity. Throughout these fluctuations in NO_3_^−^, tissue NH_4_^+^ concentrations usually remain low, indicating a tight coupling between NO_3_^−^ and NH_4_^+^ assimilation, primarily GS activity. The set points for N homeostasis are fundamentally linked to those of other elements; these are often expressed as ratios, such as C:N (reviewed by Fañanás-Pueyo et al., 2025). The C:N ratio changes in tissues during the switch from vegetative to floral ontogeny, from 15-20:1 to 80-100:1, and two conserved kinases (TOR and SnRK1) play key parts in signalling in all eukaryotes. Other elemental ratios have been studied in much less detail, for example, N:P, although these are linked to the type of plant, higher in graminoids than forbs, and linked to stress tolerance (Güsewell, 2004). Long-distance transport is also closely coupled with the need for electrical charge balance and for example, potassium is well known to counter NO_3_^−^ movement in the xylem and phloem (Drechsler et al., 2015).

## 3. Cellular homeostasis of N

**Figure 2.**
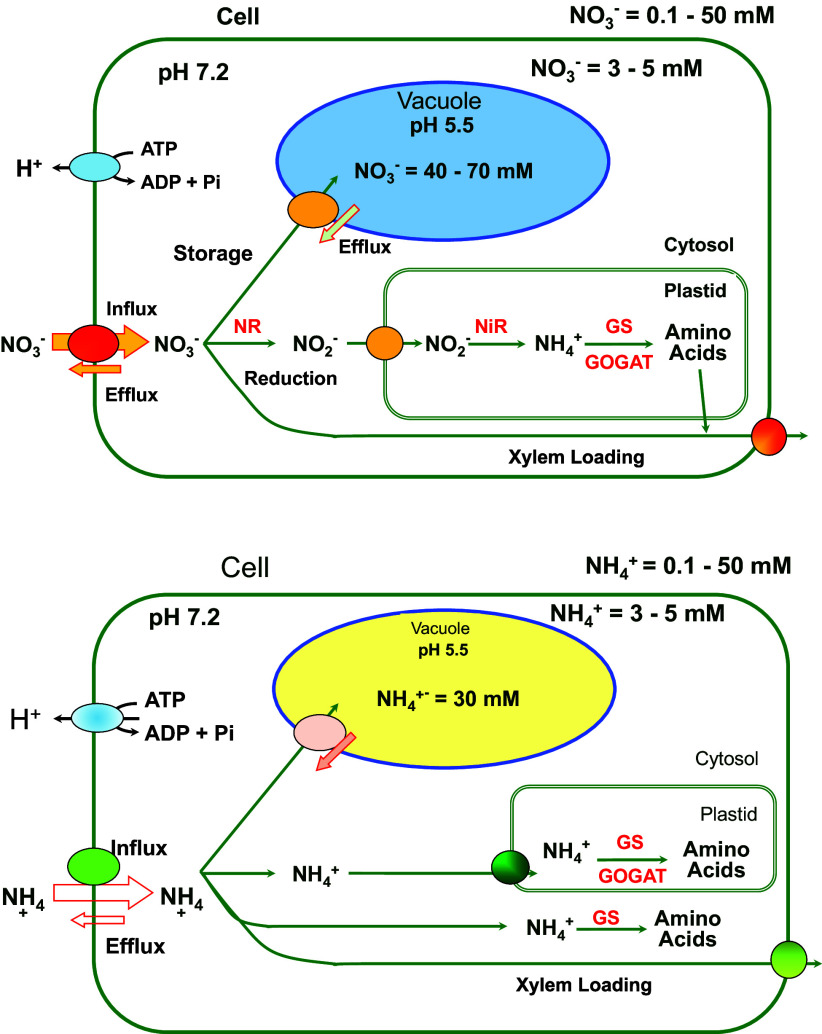
Diagrammatic overview of plant cell ammonium and nitrate homeostasis showing metabolism and transport. Upper, nitrate; Lower, ammonium.

The set points for cytosolic homeostasis of NO_3_^−^ and NH_4_^+^ seem to be similar, with both in the low mM range for the cytosol and an order of magnitude greater for the vacuole (see Figure 2). Although the published values can vary hugely over several orders of magnitude for NH_4_ *^+^* from 0.02 to 0.2 M, depending on the supply and method used (Miller et al., 2001). There can be fundamental metabolic consequences of NO_3_^−^ and NH_4_^+^ use by plant cells that could directly act on intracellular homeostasis through regulatory mechanisms for pH and redox regulation. For example, excessive NO_3_^−^ transport can acidify the cytosol and activate proton pumps to restore pH homeostasis (Feng et al., 2020). Nitrate assimilation consumes reducing power in the cell through NADH- or NADPH-mediated transfer of reductant. In Arabidopsis leaf cells, both photosynthesis and nitrate reductase activity were shown to alter the cytosolic NO_3_^−^ set point (Cookson et al., 2005). These primary assimilatory processes may provide another level of control that is important when there is an excessive supply of either of these forms of N supply.

When plants are N replete, the few measurements which have been made suggest that cytosolic NH_4_^+^ and NO_3_^−^ activities are maintained at mM activities that are independent of the external supply (e.g. NO_3_^−^: Miller & Smith, 2008; NH_4_^+^: Wells & Miller, 2000; Zhou et al., 2015). These homeostatic set point values are higher than the affinities of the enzymes that assimilate the ions; GS/GOGAT for NH_4_^+^ and NO_3_^−^ reductase for NO_3_^−^ (see Figure 2). Suggesting that assimilatory enzyme activities are not directly determining the cytosolic set points. A range of set point values has been reported, depending largely on the type of method used, and as discussed above, more measurements and new techniques are needed.

### 3.1. The evidence for set points of cellular NH_4_^+^ and NO_3_^−^

A previous review (Miller & Smith, 2008) described the evidence that cytosolic NO_3_^−^ homeostasis is regulated and has a potential role in nutrient sensing and signalling. It was suggested that root tips may be particularly important in this sensing role. Nitrate is essential for plant growth and development, even in organs like seeds where its nutritional role is unclear (Chopin et al., 2007). It can act as a signal for germination and shoot–root growth balance. Different cell types and tissues can show varying NO_3_^−^ concentrations, with vacuolar NO_3_^−^ pools acting as reservoirs to maintain cytosolic NO_3_^−^ levels. Twenty years ago, the evidence for these ideas was largely based on measurements using intracellular NO_3_^−^-selective electrodes. Nitrate-selective microelectrodes measurements have shown environmental conditions when cytosolic nitrate changed, for example, during light/dark changes in leaf cells, NO_3_^−^-starvation and when NO_3_^−^ and ammonium or glutamine were supplied together as a mixed supply of N (Miller & Smith, 2008).

The stability of cytosolic NO_3_^−^ is controversial, and even now, the methods to measure it are limited – new techniques are needed, and despite progress in using genetically encoded sensors for other ions (Sadoine et al., 2023), obtaining quantitative NO_3_^−^ measurements remains a tricky nut to crack (*Clophensor* Demes et al., 2020; *NitraMeter3.0* Chen et al., 2022; *NitrOFF* Cook et al., 2025). These genetically encoded sensors are often pH sensitive, but in the cytoplasm the tight regulation of pH should minimise this problem. The high affinity of the bacterial-derived sensors, for example, the nitrate recognition domain NreA from *Staphylococcus carnosus* (K_d_=9 μM) of *NitrOFF*, should be saturated at the mM concentrations suggested from microelectrode measurements. For a comparison of the properties of genetically encoded sensors for NO_3_^−^, see Table S1 in Cook et al. (2025).

The toxicity of NH_4_^+^ accumulation in biology is widely accepted and therefore the need for plants to regulate their cellular concentration is less controversial (Bittsánszky et al., 2015).

### 3.2. Nitrate and ammonium transporters

Four gene families have been identified as NO_3_^−^ transporters; these are proton-coupled symporters NRT1s (NPFs) and NRT2s (Wang et al., 2012), proton/anion antiporters CLCs and the NAXT/SLAC/SLAH anion channels (Kollist et al., 2011). Many NRT1 (NPF) and NRT2 NO_3_^−^ transporters have been characterised in detail, they are proton-coupled cotransporters that can operate efficiently over a large range of NO_3_^−^ concentrations (reviewed in Wang et al., 2012; Xu et al., 2012). Two families of NH_4_^+^ transporters (AMT1 and 2) are well described and, like the NRTs, also have wide-ranging affinities for NH_4_^+^ (Xu et al., 2012). The entry of NH_4_^+^ into cells can occur via non-selective cation channels, driven by the large negative membrane potential across the plasma membrane (Zhou et al., 2015). Both types of transporters, NO_3_^−^ and NH_4_^+^, have proton-coupled mechanisms to drive uptake from low external concentrations of the ions. In Figure 2, we have summarised the main cellular pathways for both ions, NO_3_^−^ and NH_4_^+^. At the cell plasma membrane, we show efflux mechanisms for both ions, but the precise genetic identity is not clear. Stretch-activated anion channels for NO_3_^−^ and outwardly directed cation channels for NH_4_^+^ have been functionally characterised (Hedrich, 2012). At the plasma membrane, nitrate efflux is mediated by AtNRT1.5,1.8,1.9 (Wang et al., 2012), SLAC/SLAH anion channels, and these are important for xylem loading and stomata guard cell function (Hedrich & Geiger, 2017). The aluminium-activated malate transporters are generally considered organic anion channels, but some family members are permeable to NO_3_^−^ (e.g. rice OsALMT7, Heng et al., 2018).

In addition to regulation of transcript levels, both types of transporters, NO_3_^−^ and NH_4_^+^, have post-translational mechanisms (e.g. phosphorylation) to regulate their activity (see Section 4) and this fact supports the view that transport is a key factor in plant N homeostasis (e.g. NO_3_^−^: Yue et al., 2025; NH_4_^+^: Wang et al., 2021).

### 3.3. Vacuolar transporters

There are three protein families involved in NO_3_^−^ transport through the vacuolar membrane, CLCs (De Angeli et al., 2006; Yang et al., 2023), NRT2s (e.g. Chopin et al., 2007) and NPFs (e.g. He et al., 2017). Plant tissue NO_3_^−^ concentrations directly follow the storage concentrations in the vacuole, which in turn generally reflect the changing levels in the soil. Remobilisation of vacuolar-stored NO_3_^−^ is important for NUE (Chen et al., 2020) and this is an important target for improving NUE that may also have consequences for water use efficiency as vacuolar NO_3_^−^ is an important osmoticum (Hodin et al., 2023). In Arabidopsis, vacuolar AtCLCa activity is regulated by phospholipids and nucleotides like ATP (Yang et al., 2023).

Information for NH_4_^+^ transport at the vacuole is complicated by the fact that NH_3_ is trapped in this acidic compartment. Ammonia, due to the pH gradient between the cytoplasm and vacuole, diffuses across the tonoplast into the acidic vacuole, where it is trapped as the charged NH^+^_4_ ion in the acid compartment. In the vacuolar membrane, the tonoplast intrinsic proteins (TIPs) that can mediate water movement have also been shown to facilitate NH_3_ transport into the vacuole (Loqué et al., 2005). Both NH_4_^+^ and NH_3_ may be exported from the vacuole with the possibility for post-translational regulation of the direction of transport. This topic is important and needs more research effort.

## 4. Regulatory network: restoration of the set point

We can accept and build on the tenet that all homeostatic control mechanisms have a minimum of three interdependent components for the variable being regulated: a receptor, a control centre and an effector. This textbook homeostatic model has been formulated for animal cells (Billman, 2020). Applying these fundamental questions to the plant cell model, we can look for the three components.

### 4.1. Cell N sensing

Are there receptors for NO_3_^−^ or NH_4_^+^ in plant cells? The transporter NRT1.1 has been described as a NO_3_^−^ transceptor linked to calcium signals via kinase activity (Wang et al., 2012). A receptor should have some capacity to specifically bind either ion with an affinity that perhaps matches the range of possible homeostatic values of the cytosolic activities of these ions. Nitrate-binding proteins that are regulators of transcription have been identified in the nucleus, for example, NLP7 (Cheng et al., 2023; Liu et al., 2022). The nucleus may seem an odd place to perceive external environmental changes in N supply, but if there is homeostasis in the cytosol and this depends on the plant’s N status, then this makes sense. The nitrate-binding domain of NLP7 has been identified and a genetically encoded fluorescent split biosensor, mCitrine-NLP7, enabled visualisation of single-cell nitrate dynamics *in planta* (Liu et al., 2022). As amino acids are the primary product of N assimilation, they have been proposed as potential negative feedback signals for N status. To this end, plant glutamate receptor-like genes have been identified as Ca^2+^ channels that bind amino acids, but their functions appear more widespread, including long-distance electrical signalling and redox sensing (Simon et al., 2023).

There are many examples of post-translational modifications of proteins involved in the transport and assimilation of NH_4_^+^ and NO_3_^−^, this tight regulation provides indirect evidence for homeostasis and set points in the N physiology.

### 4.2. Regulation of assimilation enzymes

The activity of the primary N assimilatory enzymes is carefully regulated by phosphorylation; for example, NR is regulated by phosphorylation and subsequent 14-3-3 protein binding (Lambeck et al., 2012) and GS (Finnemann & Schjoerring, 2000). These are enzymes that catalyse key steps in N assimilation and homeostasis, as the former generates toxic nitrite (NO_2_^-^) and the latter is the confluence of C and N assimilation. NR activity can generate nitric oxide (NO) when NO_2_^-^ concentrations increase, and this reactive oxygen species can result in S-nitrosylation, reacting with cysteine thiols in proteins, an indicator of stress, redox status and an imbalance in N homeostasis. This reaction can also regulate protein SUMOylation linking N status to plant immunity (Borrowman et al., 2023). This mechanism is a reversible post-translational modification where SUMO (small ubiquitin-like modifier) proteins are covalently attached to lysine residues on target proteins.

### 4.3. Regulation of NH_4_^+^ and NO_3_^−^ transporters

Phosphorylation of transporters is a common regulatory mechanism and there are many examples (Hao et al., 2023). The calcium-dependent protein kinase and CIPK families of plant protein kinases function in calcium signalling pathways and are important in the regulation of N cellular homeostasis. Both NH_4_^+^ (AMT1.1/2) and NO_3_^−^ (NRT1.1) transporter activity in the model plant Arabidopsis are regulated by the activity of CIPK23 (reviewed by Ródenas & Vert, 2020). At high NH_4_^+^ concentrations, the AMT transport activity is inactivated by phosphorylation. In contrast, at low NO_3_^−^ concentrations, AtNRT1.1 transport activity is activated by CIPK23 phosphorylation. There is allosteric regulation of plant AMTs, involving a phosphorylation switch that functions in a feedback loop to restrict NH_4_^+^ uptake (Lanquar et al., 2009).

The activity of NRT2 transporters is also regulated by phosphorylation, including their interaction with the Nar2 partner proteins that are required for function (Jacquot et al., 2020; Li et al., 2024). Taken together there are multiple levels of control for the entry of inorganic N into non-leguminous plants. In legumes, the situation is further complicated by the activity of the symbiotic bacteria living in nodules and generating NH_3_ from gaseous atmospheric N_2_.

### 4.4. Regulation of vacuolar transporters

All three protein families of vacuolar NO_3_^−^ transporters can be post-translationally regulated. In Arabidopsis, vacuolar AtCLCa NO_3_^−^ selectivity is determined by a proline residue (Wege et al., 2010) and activity is regulated by phospholipids and nucleotides like ATP (De Angeli et al., 2009; Yang et al., 2023). The vacuolar CLCs also have an important role in cytosolic pH regulation (Demes et al., 2020), which is key for pH balance during the switch between NH^+^_4_ and NO_3_^−^ external supply. This provides an interesting direct link between N supply, homeostasis and pH regulation that is worthy of future investigation. The activity of the aquaporin TIPs that facilitate NH_3_ transport (Loqué et al., 2005) has also been shown to be regulated by phosphorylation of the protein (Maurel et al., 1995). The direction of tonoplast transport of NH_4_^+^ and NH_3_ may be post-translationally regulated and vacuolar accumulation of NH_4_^+^ is regulated by CAP1 (Bai et al., 2014).

### 4.5. Transcription factors

Plant transcription factors (TFs) are important molecular regulatory systems for N homeostasis, typically comprising 5% of the genome (Blanc-Mathieu et al., 2024), they coordinate N transport and assimilation (e.g. NO_3_^−^, Sámano et al., 2024). There are several well-known examples, for example, HY5 is important for C:N partitioning (Chen et al., 2016). The NIN-like proteins (NLPs) are a group of TFs belonging to the RWP-RK gene family, they act as major nitrate sensors and are implicated in the primary NO_3_^−^ response within the nucleus of both non-leguminous and leguminous plants through their RWP-RK domains. The NLPs can act as intracellular nitrate sensors, as they can bind NO_3_^−^ and thereby alter the expression of other transcripts linked to the N status of the cell. In the model plant, Arabidopsis, there are nine *NLP* genes expressed in *Arabidopsis* shoots, and the role of some family members in binding NO_3_^−^ to alter transcription is well established (Sámano et al., 2024). In a legume, NO_3_^−^ induces SUMOylation of the NLP1 TF (Liu et al., 2026). This mechanism may yet prove to be more widely found among plants, providing a link between N homeostasis and TFs.

As TFs are more general regulators of N acquisition and homeostasis, they have been targets for efforts to improve crop fertiliser use (Maurya et al., 2020). There are now many published examples of altered expression of N-responsive TFs that have been used in crops to have significant effects on NUE (see Table 1).

**Table 1:** Some N-related TFs used in crop plants to improve NUE (redrawn from Maurya et al., 2020)

Transcription factor family	Gene	Target species	Physiological and phenotypic responses	Reference
MADS-box	*OsMADS57*	Rice	Increased expression of *OsNRT2.1/2.2/2.4* and *OsNRT2.3a* Increased root to shoot N remobilisation, Shoot-root ^15^N content ratios were increased up to 76% at low NO_3_^−^ supply	Huang et al., 2019
	*OsMADS25*	Rice	Increased biomass, PR length, lateral root no. and length in the presence of NO_3_^−^	Yu et al., 2015
NLP	*OsNLP1*	Rice	Increased growth, grain yield and NUE up to 20.5% under low NO_3_^−^ supply	Alfatih et al., 2020
	*OsNLP4*	Rice	Increased biomass, grain yield (30%) and NUE (47% moderate N supply)	Wu et al., 2021
	*ZmNLP5*	Maize	Regulates nitrite reductase expression. Knock-out accumulated less seed N.	Ge et al., 2020
b-ZIP	*TabZIP60*	wheat	Increased NADH-dependent glutamate synthase (NADH-GOGAT) activity Increased lateral root branching, N uptake and spike number and improved grain yield >25% in field trial	Yang et al., 2019
NAC	*OsNAP*	Rice	Increased N content in grain and flag leaf, higher grain weight and yield up to 10.3%. Higher seed-setting rate Delayed senescence	Liang et al., 2014
	*NAM-B1*	Arabidopsis wheat	Increase in N remobilisation from leaves to grain. Increased grain protein and mineral content ~30%	Uauy et al., 2006
	*Ta NAC2−5A*	Wheat	Increased expression of TaNRT2.3B and GS2 Increased biomass and grain yield (up to 10% at low NO_3_^−^ level). Increased root NO_3_^−^ influx rate	Li et al., 2020
MYB	*SiMYB3*	Millet, Arabidopsis and rice	Increased expression of NRT1.1, NIA2, ANR1, NLP7, LBD37, LBD38, LBD39, TAR2 and IPT3 Increased grain weight, total and seed N and root growth under low N, P and K.	Ge et al., 2019
	*OsMYB305*	Rice	Increase in expression of OsNRT2.1, OsNRT2.2, OsNAR2.1, OsNiR2, NADH-GOGAT, Pyr-K and G6PDH under low N conditions Increased tillering, shoot dry weight and total N concentration. Increased 15NO3^−^ influx	Wang et al., 2020
Dof	*RDD1*	Rice	Increased *GS1;1* expression Increased chlorophyll content NO_3_^−^ and NH_4_- uptake under limited N supply. Higher grain harvest index and yield.	Iwamoto & Tagiri, 2016
	*ZmDof1*	Rice	Increased PEPC activity and asparagine content. significant increases in the rate of photosynthesis, increases in the amounts of both N and C per seedling, increased root N and biomass under limited N supply	Kurai et al., 2011
	*ZmDof1*	Wheat and Sorghum	Increased PEPC activity and amounts of N and C per seedling. Higher biomass production under up to 59% limited N supply	Peña et al., 2017
NF-Y	*TaNFYA-B*	Wheat	Higher expression of transporters NRT1.1, NRT2.1 and some phosphate transporters. Increased Lateral root growth and root NO_3_^−^ influx. Increased tissue NO_3_^−^, grain yield spike number under differing N supply levels	Qu et al., 2015

## 5. Future perspectives and N use efficiency

**Figure 3.**
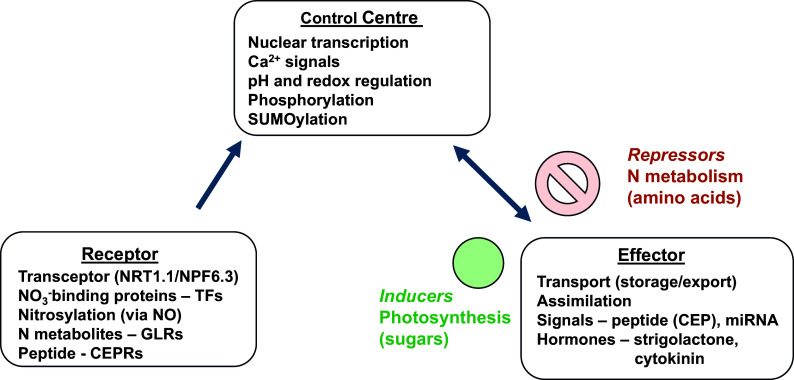
Identifying the model components of plant N homeostasis.

Improving N use efficiency (NUE) is the target of major research efforts. Plant N homeostasis and NUE are interdependent and closely linked. Altering N set points is a key component of attempts to improve NUE and here an opportunity can be provided by natural ecosystems as research using rapidly growing species adapted to nutrient-poor environments signpost the route to better crops. As described above, TFs are targets for altering C:N ratios and NUE as they offer a high tier of control (Figure 3) and there are many candidates (Table 1). It remains to be tested to what extent the effects of each TF may be additive, and this may provide a breeding opportunity. Both N assimilation and uptake are important components of NUE. The assimilation of N is limited by the photosynthetic production of sugars for amino acid synthesis by GS/GOGAT activity. The energy from carbon metabolism and supply of reductant from photosynthesis power the uptake and assimilation of N. Therefore, plant N homeostasis and NUE are directly linked to carbon assimilation and future efforts to improve crops must recognise this fact and integrate both traits into breeding programmes. The supply of N to cells, maintaining an optimal concentration for assimilation, is a fundamental driver for homeostasis both at the cellular and whole plant levels. If N homeostasis breaks down this is likely to be very detrimental for growth, but when it is sub-optimal then crop productivity is below ideal. Breeding future crops to improve the plasticity of N homeostasis is likely to be a promising strategy, particularly with the threat of climate change extreme weather events. Therefore, a better understanding of plant N homeostasis and what can limit it in cells and the whole plant is fundamentally important for improving NUE. A holistic view that includes N and C cycling is important for this progress.

In the future, a better understanding of factors extending beyond the plant, into the rhizosphere and the soil, that influence N homeostasis is important. For example, soil C supply is important for nutrient cycling and the slow release of N for crops. Maintaining a slow, steady N supply that matches the changing needs of the crops and balancing homeostasis can directly depend on root activity. Root exudates can modify soil microbial activities to influence the form of N supply and optimise growth. Optimising the balance in the N supply, NO_3_^−^ and NH_4_^+^ (Feng et al., 2020) for the crop during plant development is likely to be an important aspect of improving crop NUE. To achieve this soil delivery target, better methods for monitoring soil nutrient delivery are needed. There are also exciting opportunities for phage engineering of the rhizosphere microbiome (Quirós et al., 2023) to better optimise microbially mediated N cycling to balance the soil supply of NO_3_^−^ and NH_4_^+^ to crops. A holistic view of N homeostasis should extend beyond the whole plant and cells into the soil for improving NUE.

## Data Availability

No new data or coding was used in preparing this manuscript.

## Abbreviations and Glossary

- Aluminium-activated Malate Transporter
- Adenylate Cyclase-Associated Protein 1 (actin-binding protein)
- Calcineurin B-like protein
- C-terminally encoded peptide
- CEP Receptor
- CBL-interacting protein kinase
- Calcium-dependent protein kinase
- glutamate synthase
- glutamine synthetase
- Nicotinamide Adenine Dinucleotide
- nicotinamide adenine dinucleotide phosphate
- Nodule inception TF
- nitrite reductase.
- NIN-like proteins
- nitrate reductase
- a family of TFs
- Slow-type anion channel
- SLAC-1 homolog anion channel
- SNF1-related protein kinase 1, a key cellular energy-sensing protein kinase.
- Small Ubiquitin-like Modifier
- transcription factor – a highly conserved serine/threonine protein kinase
- Target of rapamycin
